# Epilepsy in *Onchocerca volvulus* Sero-Positive Patients From Northern Uganda—Clinical, EEG and Brain Imaging Features

**DOI:** 10.3389/fneur.2021.687281

**Published:** 2021-06-03

**Authors:** Rodney Ogwang, Albert Ningwa, Pamela Akun, Paul Bangirana, Ronald Anguzu, Rajarshi Mazumder, Noriko Salamon, Oliver Johannes Henning, Charles R. Newton, Catherine Abbo, Amos Deogratius Mwaka, Kevin Marsh, Richard Idro

**Affiliations:** ^1^College of Health Sciences, Makerere University, Kampala, Uganda; ^2^KEMRI-Wellcome Trust Research Programme, Centre for Geographic Medicine Coast, Kilifi, Kenya; ^3^Centre of Tropical Neuroscience, Kitgum, Uganda; ^4^Division of Epidemiology, Medical College of Wisconsin, Institute for Health and Equity, Milwaukee, WI, United States; ^5^Department of Neurology, University of California, Los Angeles, Los Angeles, CA, United States; ^6^Department of Radiological Sciences, David Geffen School of Medicine, University of California, Los Angeles, Los Angeles, CA, United States; ^7^Division of Clinical Neuroscience, The National Centre for Epilepsy, Oslo University Hospital, Oslo, Norway; ^8^Department of Psychiatry, University of Oxford, Oxford, United Kingdom; ^9^Nuffield Department of Medicine, Centre for Tropical Medicine and Global Health, University of Oxford, Oxford, United Kingdom

**Keywords:** seizures, MRI, EEG, onchocerciasis, epilepsy

## Abstract

Globally, epilepsy is the most common chronic neurological disorder. The incidence in sub-Saharan Africa is 2-3 times higher than that in high income countries. Infection by *Onchocerca volvulus* may be an underlying risk factor for the high burden and based upon epidemiological associations, has been proposed to cause a group of disorders—*Onchocerca* associated epilepsies (OAE) like nodding syndrome (NS). To improve our understanding of the disease spectrum, we described the clinical, electroencephalographic (EEG) and magnetic resonance imaging (MRI) features of children with epilepsy and sero-positive for *Onchocerca volvulus* (possible OAEs other than nodding syndrome). Twenty-nine children and adolescents with non-nodding syndrome OAE in northern Uganda were enrolled. A diagnosis of OAE was made in patients with epilepsy and seizure onset after age 3 years, no reported exposure to perinatal severe febrile illness or traumatic brain injury, no syndromic epilepsy diagnosis and a positive Ov-16 ELISA test. Detailed clinical evaluation including psychiatric, diagnostic EEG, a diagnostic brain MRI (in 10 patients) and laboratory testing were performed. Twenty participants (69%) were male. The mean age was 15.9 (standard deviation [SD] 1.9) years while the mean age at seizure onset was 9.8 (SD 2.9) years. All reported normal early childhood development. The most common clinical presentation was a tonic-clonic seizure. The median number of seizures was 2 (IQR 1–4) in the previous month. No specific musculoskeletal changes, or cranial nerve palsies were reported, neither were any vision, hearing and speech difficulties observed. The interictal EEG was abnormal in the majority with slow wave background activity in 52% (15/29) while 41% (12/29) had focal epileptiform activity. The brain MRI showed mild to moderate cerebellar atrophy and varying degrees of atrophy of the frontal, parietal and occipital lobes. The clinical spectrum of epilepsies associated with Onchocerca may be broader than previously described. In addition, focal onset tonic-clonic seizures, cortical and cerebellar atrophy may be important brain imaging and clinical features.

## Introduction

Epilepsy is the leading chronic neurological disorder affecting over 50 million people globally (1, 2). The burden is highest in low and middle-income countries (LMIC) where ~80% of cases live (3). The risk of premature death in people with epilepsy is up to three times higher than in the general population. Importantly however, ~70% of people with epilepsy could live seizure-free if properly diagnosed and treated (4). Low-income countries, particularly, within sub-Saharan Africa, report the highest burden of symptomatic epilepsies (5–7) in addition to a high treatment gap estimated at 68.5% (8). Furthermore, these individuals and their families suffer from high levels of stigma and discrimination (4).

Recently, it has been proposed that infestation by the filarial worm, *Onchocerca volvulus*, may be epileptogenic (9–11). *O. volvulus*, is a filarial nematode transmitted by the black fly (Simulium spp.) (12). It is known to cause human Onchocerciasis, a highly debilitating disease, primarily characterized by visual impairment and skin dermatosis (13). It is endemic in sub-Saharan Africa, Latin America and Yemen, where an estimated 20.9 million people are affected. More recently, childhood epilepsy has been suggested as a formerly unrecognized but potentially associated clinical manifestation (11, 14).

Initial reports of a possible association between infection by *O. volvulus* and epilepsy came from Mexico (15). Further evidence has emerged from several independent surveys in west, central and east African countries demonstrating an increasingly higher burden of epilepsy in regions with increasing community microfilariae load (16–19). A meta-analysis of these surveys demonstrated that the prevalence of epilepsy in *O. volvulus* endemic regions increased, by ~0.4% for every 10% increase in the prevalence of onchocerciasis in the community (20). Studies of nodding syndrome, a poorly understood childhood epileptic encephalopathy, following epidemics in northern Uganda and South Sudan, further strengthened the potential aetiological link between epilepsy and *O. volvulus* (21–24). Similarly, reports of Nakalanga syndrome, another neurological disorder characterized by severe stunting with or without epilepsy and occurring solely in regions endemic for *O. volvulus* further supported a possible aetiological role of this parasite in neurological diseases (25, 26). Based upon epidemiological evidence the term Onchocerca associated epilepsies (OAE) was later coined to describe a spectrum of epilepsy disorders that maybe linked to infection with the filarial worm, *O. volvulus* (11). However, it is noteworthy that the epileptogenic nature of *O. volvulus* infection remains inconclusive. Therefore, it is possible that other indirect mechanisms may explain the observed association (27, 28).

The clinical descriptions of neurological disorders that have been associated with *O. volvulus* (Nodding syndrome and Nakalanga syndrome) suggest that these disorders develop in normally developing children between the ages of 3–18 years and present with varied degrees of symptoms which include: generalized seizures, cognitive decline, stunting, delayed sexual development in addition to psychiatric and psychological difficulties (21, 24, 29, 30). In nodding syndrome, a distinctive pathognomic feature—bouts of repeated head nods precipitated by food or cold weather develops early in the disease (31, 32). Head nodding is associated with varied degrees of the above mentioned OAE symptoms (32). In Nakalanga syndrome, the main feature is severe growth retardation (25, 26, 30). Especially in Nodding syndrome, disease severity varies with the intensity of onchocerca exposure and or the microfilaria load (33). For example, the disease symptoms in South Sudan and northern Uganda that report a higher prevalence of *O. volvulus* are more severe than that observed in southern Tanzania (29). The complete clinical description of the spectrum of epilepsies possibly associated with *O. volvulus* remains unclear.

It has been thought that (1) infection with *O. volvulus* is associated with a spectrum of neurological diseases; (2) patients with milder neurological involvement majorly develop epilepsy while those with more severe disease develop nodding syndrome or the Nakalanga syndrome (34–36). We aimed to describe the clinical, EEG, and brain imaging features of children diagnosed with an epilepsy possibly associated with Onchocerca (OAEs other than Nodding or Nakalanga syndromes).

## Materials and Methods

### Study Design

This case series describes the clinical, electrophysiological, and brain imaging features of children with an epilepsy possibly associated with *O. volvulus*. The study was nested within an ongoing case control study investigating the pathogenesis of nodding syndrome. In the main study, 154 patients with nodding syndrome were consecutively recruited and compared to two sets of controls: 154 controls with non-nodding syndrome epilepsy and 154 normal sibling controls. The objective of the parent study was to determine if cross reacting antibodies were the underlying pathogenic mechanism in nodding syndrome.

### Setting

The study was conducted in Kitgum General Hospital (KGH) between 2017 and 18. KGH is a 100-bed facility in northern Uganda. This region of northern Uganda is populated primarily by the Acholi, a Luo speaking people. It has intense malaria transmission (37), is endemic for *O. volvulus* and has a remarkably high burden of epilepsy (38). The vegetation is Savanah with many swamps and fast flowing rivers (39). From the early 2000's, the region suffered an epidemic of the nodding syndrome which was described within the districts of Kitgum, Pader, Gulu. In these times, almost 95% of individuals in the area were displaced persons because of the cruel civil war waged by the Lord Resistance Army (LRA) against the Ugandan Government. KGH is the nodding syndrome and epilepsy treatment and referral center in the region (40).

### Participants

Participants were patients defined to have an *O. volvulus* associated epilepsy (OAE). The inclusion criteria were; (1) epilepsy with seizure onset between the ages of 3 and 18 years; (2) no reported developmental difficulties before the onset of epilepsy; (3) no obvious cause for the epilepsy on history or clinical exam, (4) geographical clustering of other persons with epilepsy in the village; (5) never diagnosed with nodding syndrome and (6) positive test for anti OV-16 serum IgG antibodies (11).

### Ethical Approval and Informed Consent Procedures

Ethical approval for the study was obtained from Makerere University School of Medicine Research and Ethics Committee (SOMREC) (REC Ref: 2015-146) and University of Oxford Tropical Research Ethics Committee (OXTREC) (Ref: 12-16). Regulatory approval was provided by The Uganda National Council for Science and Technology (Ref: HS-1991). Parental consent alongside an assent was obtained from each participant.

### Study Measurements and Procedures

#### Clinical Assessment

Participants were recruited consecutively from the epilepsy treatment centers in the nodding syndrome affected districts of Kitgum, Pader, and Lamwo. All received a standardized clinical assessment, reporting the clinical history (including psychiatric) and physical exam. The history included the child's development from pregnancy to the onset and the progressive development of symptoms. The physical examination included general, nutritional, and neurological assessments, and a description of the burden and types of seizures. Nutritional status was assessed against WHO 2000 anthropometric standards. The Child and Adolescent Symptom Inventory-5 (CASI-5) was used to document psychiatric disorders classified according to the DSM-5 criteria.

#### Laboratory Procedures

Ten milliliters of blood were drawn from each participant as part of standard of care testing. This consisted of a full blood count, liver and renal function tests. The presence of malaria was determined using the *Plasmodium falciparum* malaria histidine rich protein-2 (HRP2) rapid diagnostic test (CareStart, 2016), while HIV infection was tested using a standard of care HIV algorithm. All participants had skin snip biopsy taken from the iliac crest. This was incubated for 48 h in normal saline and the sediment examined under a microscope for *O. volvulus* microfilaria. Following a previously published protocol (41, 42), Onchocerca exposure was determined by assessing for the presence of anti-OV-16 serum IgG4 antibody levels using previously described ELISA assays (41).

#### Neurophysiology and Imaging

All participants underwent a 30 min electroencephalogram recording using a Neuro-Travel light-EEG machine, version 2.12.106. Standard silver/silver chloride (Ag/AgCl) cup electrodes were attached to the scalp according to the international 10/20 system. Recordings with 19 channels were performed in awake state for 30 min, including intermittent photic stimulation and hyperventilation. EEG recordings were reviewed blinded by a board-certified neurophysiologist (O.J.H.).

Participants (1 in 3) were randomly selected for MRIs of the brain (10 participants) and spinal cord (3 of the 10 participants). The MRIs were obtained using a 1.5T Achieva MRI (Philips Medical Systems). Anatomical MRI, including pre- and post-gadolinium contrast T1-weighted images, T2-weighted images, fluid-attenuated inversion recovery (FLAIR) images, diffusion-weighted images and susceptibility weighted images were collected. MRI was evaluated by an experienced neuro-radiologist (N.S.) blinded to the clinical status of the subjects.

### Data and Statistical Analysis

All data was collected using standardized case record forms and entered into an Epi Info™ version 7.1.5. Data were compiled using Microsoft Excel 2016 before being exported to STATA V.12.0 (StataCorp, Texas, USA) and GraphPad Prism V.6.01 (GraphPad Software, California, USA). CASI-5 items were scored using the symptom count cut-off score method which indicates whether child/adolescent has the prerequisite number of symptoms necessary for a DSM-5 diagnosis. Continuous data was summarized as means (standard deviation [SD]) or median (inter-quartile range [IQR]) where appropriate. Categorical data was summarized as proportions and percentages.

## Results

### Demographic Characteristics

**Figure 1:**
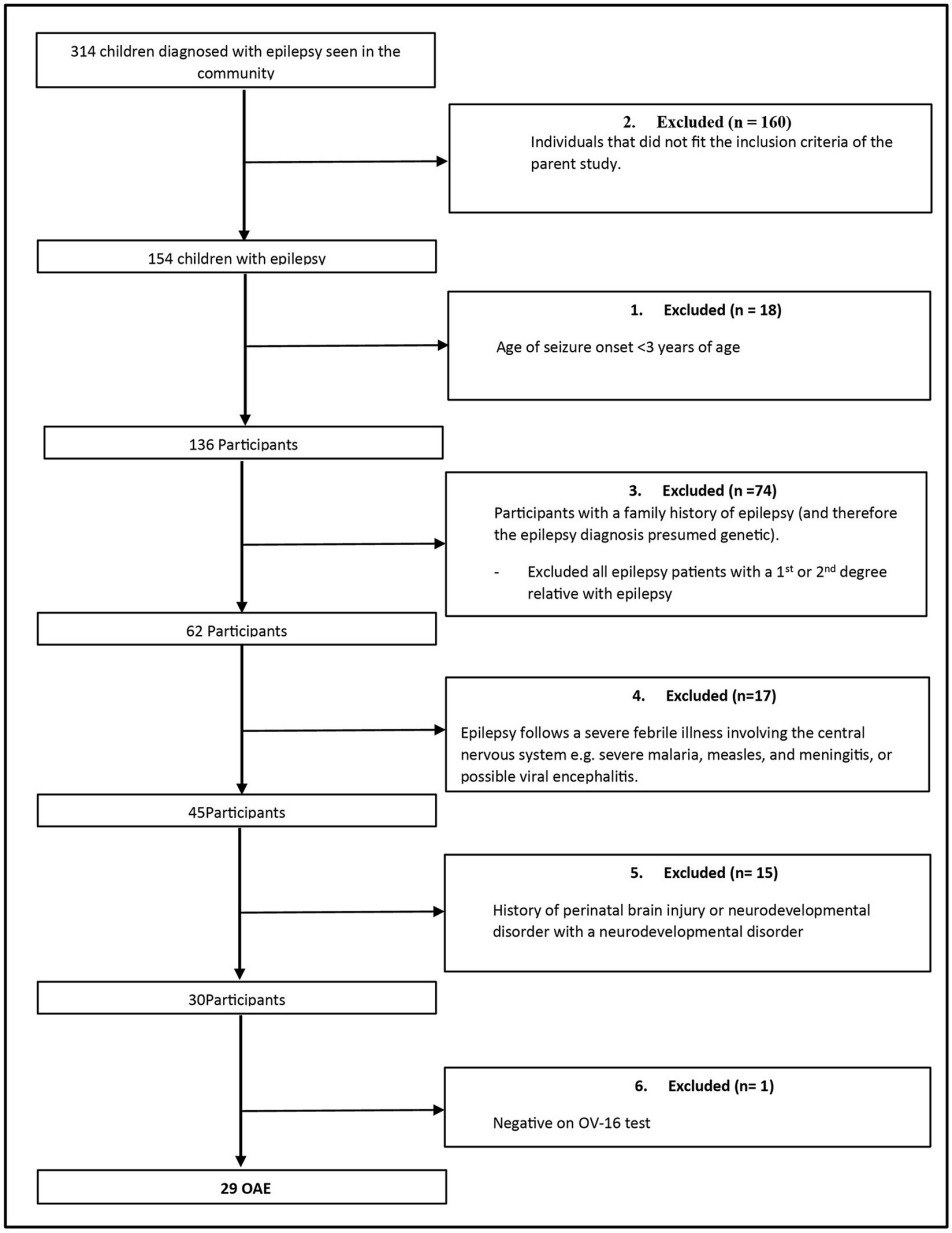
Participant selection.

A total of 17 epilepsy outreach clinics in the three districts were visited. Three hundred and fourteen consecutive patients with non-nodding syndrome epilepsy were screened for inclusion into the main study as epilepsy controls. One hundred and fifty-four individuals were selected to participate in the parent study. Of these, 29 (18.8%) fulfilled the criteria for an OAE (Figure 1).

The mean age was 15.9 (SD1.4) years and 20 (68.9%), were male. The average age at seizure onset was 9.8 (SD 2.9) years. Among the participants, 28 (97%) reported previously living in an internally displaced people's (IDP) camp as they escaped from the Lord's Resistance Army insurgency. Most dropped out of school with 19 (66%) citing epilepsy as the reason. There was no significant difference in the demographic features between children categorized as having an OAE and other persons with epilepsy (PWE) (Table 1).

**Table 1:** Patient characteristics.

	**OAE (*n* = 29)**	**PWE (*n* = 125)**	***P*-value**
**Demographic features**			
Age in years, mean (SD)	15.9 (1.93)	15.41 (1.99)	0.18
Sex, male *n* (%)	20 (68.9)	68 (54.4)	0.15
Parent status alive *n* (%)	20 (68.9)	86 (68.8)	0.98
Age of seizure onset (years) mean (SD)	9.8 (2.9)	8.5 (4.0)	0.11
Lived in an IDP camp *n* (%)	28 (96.5)	119 (92.2)	0.75
Years spent in IDP camp mean (SD)	6.0 (3.0)	5.1 (2.6)	0.13
**Education level**			
Dropped out of school *n* (%)	19 (65.5)	81 (64.8)	0.94
Highest level achieved at point to study *n* (%)			
Nursery		1 (0.8%)	
Primary	29(100%)	114 (91.2%)	
Secondary		4 (3.2%)	
Missing		6 (4.8%)	
**Anthropometric measurements**			
Mid Upper Arm Circumference, cm, mean (SD)	24.0 (3.9)	24.0(15.2)	0.98
Height for age z score, mean (SD)	−1.33 (1.21)	−1.03 (1.06)	0.18
Proportion under height (z score ≤ −2), *n* (%)	8 (27.5%)	27(21.6)	0.47
Weight for age z score, mean (SD)	−1.67 (1.29)	−1.69 (1.22)	0.96
Proportion underweight (z score ≤ −2), *n* (%)	10 (34.5%)	47(37.6%)	0.83
Body Mass Index for age z score, mean (SD)	−1.28 (1.59)	−1.47(1.16)	0.54
Proportion undernourished *n* (%)	7 (24.1%)	37(29.6%)	0.65
Heart rate, beats per minute, mean (SD)	82.03 (13.51)	86.2 (14.61)	0.16
Systolic blood pressure, mmHg, mean (SD)	114.4 (12.48)	114.3 (15.33)	0.95
Diastolic Blood Pressure, mmHg, mean (SD)	72.48 (10.24)	74.41(11.20)	0.39
**Seizures**			
Seizure free *n* (%)	6 (20.6%)	23 (18.4%)	0.77
**Seizure types**			
Tonic-clonic *n* (%)	26 (89%)	116(92.8%)	0.56
Focal unaware seizure with behavior arrest *n* (%)	7 (24%)	26 (20%)	0.69
Myoclonic *n* (%)	0(0%)	0(0%)	1.0
**Psychiatric disorder (CASI- 5 care giver reported)**			
**Behavioral disorders**			
Oppositional defiant disorder *n* (%)	0 (0.0%)	16 (12.8%)	0.04
Conduct disorder *n* (%)	0 (0.0%)	0 (0.0%)	1.0
Attention deficit hyperactivity disorder *n* (%)	0 (0.0%)	18 (14.4%)	0.02
**Emotional disorder**			
Generalized anxiety disorder-A *n* (%)	5 (17.2%)	18 (14.4%)	0.77
Panic Disorder *n* (%)	1 (3.5%)	2 (1.6%)	0.46
Social anxiety disorder *n* (%)	0 (0.0%)	2 (1.6%)	1.0
Specific Phobia *n* (%)	2 (6.9%)	17(13.6%)	0.53
Separation anxiety disorder *n* (%)	2 (6.9%)	13 (10.4%)	0.74
Major depressive disorder *n* (%)	1 (3.5%)	21(16.80)	0.07
Now persistent depression (dysthymia) *n* (%)	1 (3.5%)	0 (0.0%)	0.18
Internalizing disorder *n* (%)	5 (17.2%)	32 (24.60%)	0.47
Externalizing disorder *n* (%)	2 (6.9%)	24 (19.20%)	0.17
Post-Traumatic stress disorder *n* (%)	1 (3.5%)	4 (3.2%)	1.0

### Clinical Features of Non-nodding Syndrome OAE

#### Neurological, Cognitive, and Behavioral Features

##### Seizures

Tonic-clonic seizures were the predominant seizure type experienced, reported in 90% (26/29) of participants. However, parents of seven participants reported observing periods of blank stares like probable focal unaware seizures with behavior arrest. No myoclonic seizures were reported.

Among participants experiencing a seizure, 21% (6/29) reported visual hallucinations, and 55% (16/29) had loss of awareness during the seizure. Participants experienced a median 2 (IQR 1–4) seizures in the past month. However, several participants reported over 10 seizures a month. Only 6 were seizure free at the time of the study.

##### Musculoskeletal Findings

There were no significant musculoskeletal changes observed among the participants. Peripheral muscle wasting of both limbs was observed in 7% (2/29). Generalized wasting or other physical deformation such as kyphosis and pectus were not observed. However, lip deformations were noted in three participants.

##### Other Neurological Complications

All had full alertness at the point of observation. No participant reported any vision, hearing, and speech difficulties. All had a normal gait and muscle tone in all limbs; however, tendon reflexes were abnormal in 21% (6/29) participants but no consistent pattern. Hyporeflexia was observed in two cases and hyperreflexia in two children. Another two cases had abnormal tendon reflexes in both limbs. There were no other motor abnormalities or obvious cranial nerve palsies were observed in any of the participants.

##### Behavioral and Psychiatric Features

The Child and Adolescent Symptom Inventory-5 (CASI-5) was used to evaluate the prevalence of behavioral and psychiatric features among the children. Compared to children with epilepsy (PWE), there were no specific behavioral disorders reported among OAE patients. However, emotional disorders, such as generalized anxiety, were reported in 17% (5/29) and internalization disorder observed in 17% (5/29) (Table 1).

##### EEG

**Figure 2:**
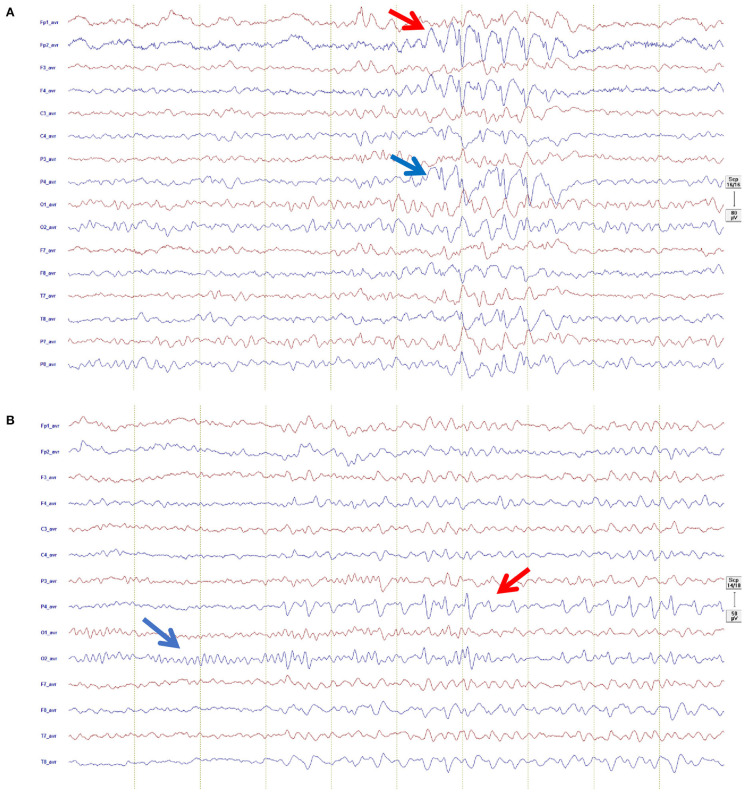
Representative EEG recordings from possible OAE epilepsy cases: **(A)** Shown in an average montage (left channels red, right channels blue, Filter 0.53–83 Hz, sensitivity 80 μ/cm, 1 sek/30 mm), showing focal epileptiform activity in form of spike-wave predominantly right frontal (red arrow) propagating to right parietal (blue arrow): **(B)** Shown in an average montage (left channels red, right channels blue, Filter 1.59–20 Hz, sensitivity 80 μ/cm, 1 sek/30 mm), showing normal posterior dominant rhythm (blue arrow) and train of right sided focal epileptiform activity in form of sharp-slow-wave predominantly in the right parietal region (red arrow).

EEG recording were not available for two individuals. The 30 min diagnostic EEG was abnormal in 63% (17/27) of individuals with OAE (Figure 2). The median (IQR) background activity was 9 (8–10) Hz. In nearly half of the recordings, 52% (14/27), we found slow background EEG activity. Five participants had focal slowing while it was multifocal in five, generalized in three, and bilateral in one participant. Among individuals with focal slowing, left-hemispheric lateralization was observed in (2/5), while Localization was frontal (2/5); occipito-temporal (2/5) and parietal (1/5).

Focal interictal epileptiform activity was observed in 41% (11/27). Generalized epileptiform activity was not found in any of the subjects. The location of the interictal epileptiform discharges (IED) were as follows: multifocal (2/11), bilateral (3/11), and right temporal (2/11) and left temporal (4/11). IED (high amplitude spikes or sharp waves) occurred with varying frequency (1 every 30 s to over one in 15 min).

##### Brain Imaging

**Figure 3:**
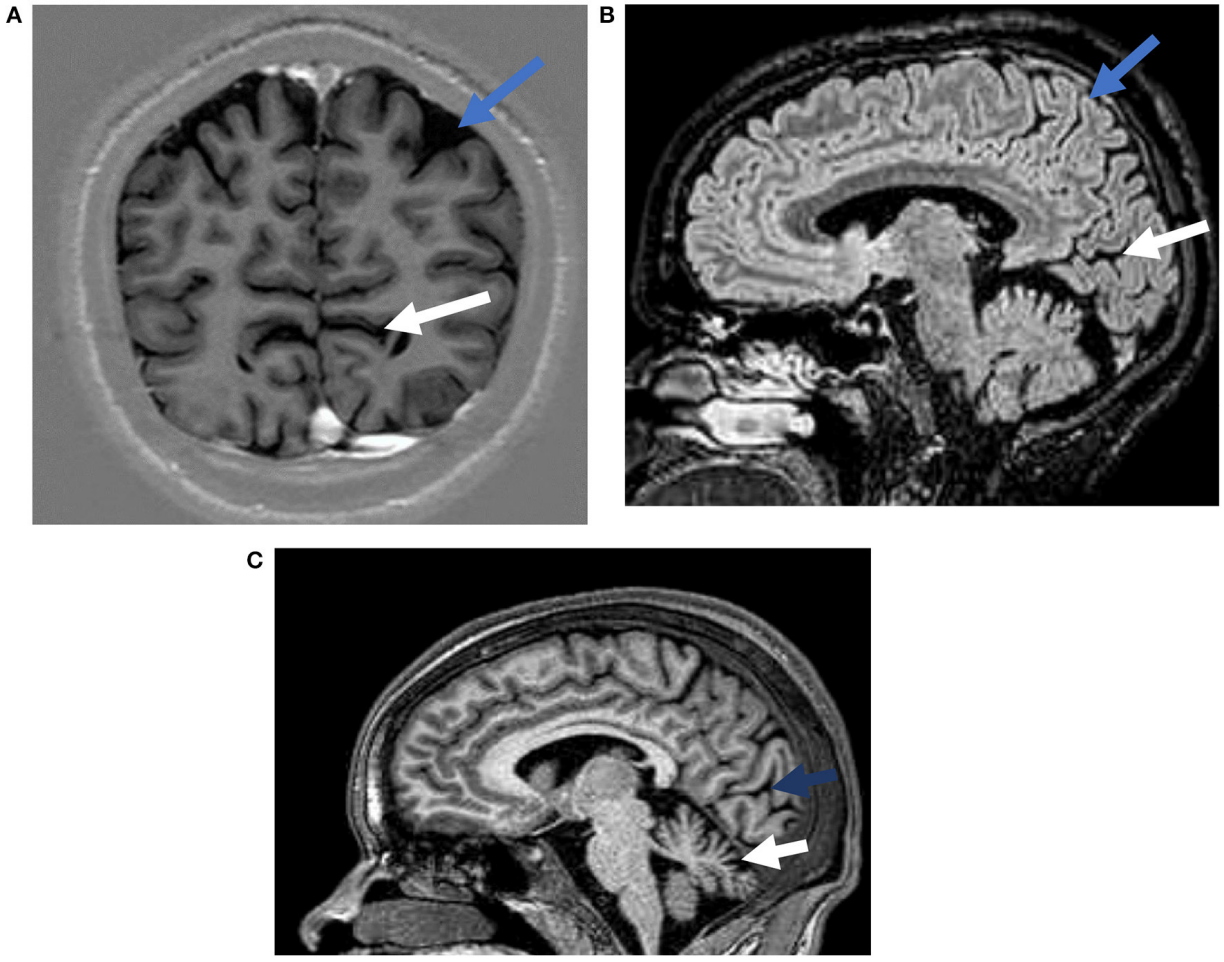
Representative magnetic resonance images from possible OAE epilepsy cases. **(A)** A T1 weighted inversion recovery image demonstrating occipital atrophy (white arrow) and parietal lobe atrophy (blue arrow). **(B)** A T2 weighted FLAIR image demonstrating occipital (white arrow), and parietal atrophy (blue arrow). **(C)** A T1 weighted image showing occipital (blue arrow), and cerebellar (white arrow) atrophy.

Ten randomly selected participants (1 in 3) underwent brain MRI with and without gadolinium contrast using a 1.5 Tesla machine (Philips Achieva). Of these, 3 also had spinal MRI. Most participants (8/10) had structural abnormalities on the brain MRI (Table 2 and Figure 3). The commonly observed abnormalities were mild to moderate cerebellar atrophy (6/10) and regional cerebral atrophy (5/10). Cerebral atrophy was noted in the following regions: bilateral medial parieto-occipital (2/10), bilateral fronto-parietal (1/10), bilateral parietal (1/10), bilateral medial occipital (1/10). No hippocampal atrophy or hippocampal sclerosis was observed. Enhancement with gadolinium did not result in any additional findings. MRI analysis of spinal cord revealed abnormalities in one participant who had atrophy of the thoracic cord without any associated signal change.

**Table 2:** Summary of brain imaging and EEG features of children who received an MRI recording.

**Participant**	**Sex**	**Seizure History**		**MRI features**		**EEG features**		
		**Estimated age of first convulsion (years)**	**Seizure type experienced**	**Brain imaging features**	**Spinal cord MR features**	**Background EEG activity frequency (Hz)**	**Any Slowing on EEG**	**Interictal epileptiform activity**
1	Female	11	Tonic-clonic	Mild to moderate cerebellar atrophy	Global atrophy of the thoracic cord without signal change	9	Yes, Generalized	Yes, Focal- left sided
2	Male	12	Tonic-clonic	Cerebellar atrophy, bilateral fronto-parietal atrophy	Normal spinal cord	7	Yes, Generalized	None
3	Male	10	Tonic-clonic	Mild medial Parieto-occipital atrophy	ND	NA	NA	NA
4	Male	4	Focal unaware seizure with behavior arrest	Normal	ND	11	Yes, Focal- right frontal	Yes, Focal- right sided
5	Male	10	Tonic-clonic and focal unaware seizure with behavior arrest	Mild cerebellar atrophy, Mild bilateral medial parieto-occipital atrophy	Normal spinal cord	9	Yes, Multifocal	Yes, Multifocal
6	Female	14	Tonic-clonic and focal unaware seizure with behavior arrest	Bilateral Parietal atrophy, Mild cerebellar atrophy	ND	10	No slowing	None
7	Male	12	Tonic-clonic	Mild cerebellar atrophy	ND	9	Yes, Bilateral occipital	None
8	Male	11	Tonic-clonic	Normal, cavum septum pellucidum	ND	8	No slowing	None
9	Male	10	Tonic-clonic and focal unaware seizure with behavior arrest	Moderate cerebellar atrophy, Moderate bilateral medial occipital atrophy	ND	9	No slowing	None
10	Male	11	Tonic-clonic	Normal	ND	11	No slowing	None

*ND, Not done; NA, not available*.

### Laboratory Findings

The mean hemoglobin was 12.7 (range 11.7–13.9) g/dl. The total white blood cell and platelet counts were normal. The liver and renal functions were normal. All participants were HIV negative, while malaria was reported in 62% (18/29) individuals. All had a positive OV-16 ELISA with a signal to noise above 2 and furthermore 2 had a positive skin snip result with observed microfilaria on skin snip biopsy.

### Treatment

All epilepsy patients received occupational (focused on burn prevention), physiotherapy, and drug adherence counseling. Any malnourished children received nutritional rehabilitation using Ready to Use Therapeutic Foods (Plumpy'Nut, Nutriset, Malaunay) and locally available food. Antiepileptic drug doses were optimized to recommended and appropriate daily doses depending on current AED regimen and seizure history. In addition, folic acid 5 mg once a day was administered. Carbamazepine was the major anti-seizure medication (ASM) used and was reported in 76% (22/29) cases. Other AEDs used included Phenytoin in 14% (4/29), Sodium valproate in 7% (2/29) and clonazepam in 4% (1/29). All participants were referred to outpatient epilepsy clinics for monthly refills.

### Outcomes

Participants were followed up and clinical evaluations repeated at 12 months after baseline examinations. At the 12 months evaluation, fifteen participants had not experienced any convulsive seizures in the previous 30 days while among those with seizures, the median (IQR) number of seizures experienced in the past month was 1(0–3). In addition, three participants experienced an episode of status epilepticus during the intervening period and were admitted at Kitgum General Hospital, two female participants became pregnant and three experienced burns.

## Discussion

Here, we described the clinical, EEG, and brain imaging features of children with epilepsies possibly associated with *O. volvulus* infection (other than nodding and Nakalanga syndromes). The study suggests that the clinical spectrum of epilepsies probably associated with *O. volvulus* infection maybe broader than previously described. In addition, it is possible that focal onset epilepsy with tonic-clonic seizures and cortical and cerebellar atrophy, maybe features associated with this unique group of epilepsies.

The broader features of OAEs may range from a severe debilitating condition as observed in patients with Nodding or Nakalanga syndromes (severe debilitation, stunting, delayed development of sexual characteristics, in addition to psychiatric, psychological and cognitive decline) (23, 24, 32, 33) to a milder condition (limited to no behavioral, physical, and psychiatric abnormalities) easily classified as epilepsy. Among nodding syndrome cases, disease progression has been documented (32, 43) but this description did not observe any significant progressive worsening of clinical symptoms. This is possibly due to the epilepsy outreach clinics that offer treatment to participants in their villages. Furthermore, within the region with ending of the decade old war, there has been improved sanitation, nutrition, vaccination together with removal of IDP camps, all considered significant risk factors of OAEs (44).

Some interictal electrophysiological features observed here appear similar to EEG features seen in nodding syndrome for example the focal or multifocal slowing of the background EEG. This was noted in 52% of the subjects in this study. Other features are distinct from nodding syndrome. Most EEGs in our study were abnormal and nearly 50% showed interictal focal epileptiform activity while interictal EEG in nodding syndrome commonly shows generalized slow spike-and-wave discharges (24). This difference, however, does not exclude the possibility that the two entities of nodding syndrome and OAE are within the same spectrum of disorder with a shared etiology of their pathogenesis. Such a wide spectrum of epileptic disorders has ever been defined as in the case of SCN1A mutations where the electroclinical entity ranges from severe epileptic encephalopathy with generalized electrographic discharges to focal seizures with febrile illness (45). It is also important to note that the majority of the participants in this study were receiving the anticonvulsant carbamazepine which may have confounded the EEG recordings (46) and show slowing of the background.

Our study found bi-hemispheric regional cortical atrophy in OAE suggesting focal onset seizures. In this cross-sectional study however, it is unclear if the regional cortical atrophy is the cause or the consequence of epilepsy. Importantly, previous longitudinal studies in focal epilepsies have found progressive cerebral atrophy associated with focal epilepsy pronounced ipsilateral to the epileptogenic zone, or extended beyond the immediate epileptic focus to involve the contralateral hemisphere (47). Future longitudinal studies are needed to evaluate the changes associated with the epileptic network in OAE that leads to cortical atrophy. Interestingly, 8 of the 10 participants imaged were found to have cerebellar atrophy, a common feature also observed in nodding syndrome. Similar to the cortical atrophy seen in this study, we are unable to assess the causal relationship of the cerebellar atrophy with OAE. The possibility of non-specific treatment related changes leading to cerebral and cerebellar atrophy cannot be ruled out. For example, long term use of Phenytoin is associated with cerebellar atrophy (48). In this study chronic use of Phenytoin was observed in aproportion of patients. Moreover, studies involving other types of epilepsies have found that unilateral focal epilepsy could lead to bilateral cerebellar atrophy and also longer duration of epilepsy was associated with cerebellar volume loss (49). Given the small number of cases in our cross-sectional study, it is difficult to assess if these clinical features (i.e., duration of disease and anti-seizure medications) led to the atrophic changes. Furthermore, our imaging studies did not find any enhancement with gadolinium. This may argue against an active breakdown of the blood-brain barrier, as seen with inflammatory processes. Also, there were no imaging evidence or signs of sequalae of prior cerebral infection or vasculopathy caused by infection.

The African epilepsy burden is estimated to range from 2.2 to 58 per 1,000 population (50). Strikingly, in *O. volvulus* endemic areas, by 2015, ~ 128,000 years of life lived with disability (YLD) where are estimated to be attributed to OAE. This may amount to over 12.5 million dollars' worth of AEDs (51). It remains inconclusive whether infection with *O. volvulus* is epileptogenic and requires further study. However, a recent study estimated that with the appropriate control interventions over 400,000 cases of epilepsy may have been prevented (14, 52). Furthermore, the impact of *O. volvulus* infection on the clinical, EEG and MRI features in children with a pre-existing epilepsy condition remains largely unexplored. Until mechanistic evidence is demonstrated, it remains vital to ensure the implementation of *O. volvulus* elimination strategies such as community-directed treatment with ivermectin (CDTi), aerial spraying and larvicidal to eradicate black flies, and finally epilepsy surveillance in onchocerca endemic areas. These activities may have the advantage of improving the livelihood of children and adolescents living within these areas. In addition, careful implementation of clinical, social and anti-seizure medication support should be provided to children diagnosed with a seizure related disorder.

The study had some limitations. First, the role of *O. volvulus* in the cause of epilepsy is unclear. Here, we used a stringent selection strategy to identify individuals likely to have an epilepsy associated with *O. volvulus*. It is possible that within the selection process several individuals may have been misclassified as either PWE or OAE leading to an under or over estimation of the OAE population. However, we believe that this strengthens the clinical description of OAE features presented. Second, the sample size of OAE participants was small, a larger study should be conducted in the future to describe the clinical spectrum of OAE features more extensively. Third, seizure history was obtained primarily from parental descriptions and may have been confounded by recall bias. Finally, only 30 min diagnostic EEGs were performed and not a prolonged recording important for investigating seizure. Finally, the study presents no CSF findings. As auto-antibodies are proposed as the pathological link between epilepsy and onchocerca infections future studies should be designed to investigate this further.

## Conclusion

The clinical spectrum of seizure disorders associated with onchocerca infections may be broader than previously described. Further from the small number of selected individuals in the present study, we propose that focal onset tonic-clonic seizures in addition to cortical and cerebellar atrophy maybe key features. A larger well-designed study maybe more informative to elucidate the clinical spectrum of OAEs.

## Data Availability Statement

The raw data supporting the conclusions of this article will be made available by the authors, without undue reservation.

## Ethics Statement

The studies involving human participants were reviewed and approved by Makerere University School of Medicine Research and Ethics Committee (SOMREC-REC Ref: 2015-146) and University of Oxford Tropical Research Ethics Committee (OXTREC) (Ref: 12-16). Regulatory approval was provided by The Uganda National Council for Science and Technology (Ref: HS-1991). Parental consent alongside an assent was obtained from each participant. Written informed consent to participate in this study was provided by the participants' legal guardian/next of kin.

## Author Contributions

RO and RI conceived the idea, conducted analyses, and drafted the manuscript. AN, PA, and RA collected field data. NS and RM conducted brain imaging. OH conducted EEG analyses. CN, CA, AM, and KM critically reviewed the manuscript. All authors have read and approved the manuscript.

## Conflict of Interest

The authors declare that the research was conducted in the absence of any commercial or financial relationships that could be construed as a potential conflict of interest.
